# A cross-cohort analysis of dental plaque microbiome in early childhood caries

**DOI:** 10.1016/j.isci.2024.110447

**Published:** 2024-07-04

**Authors:** Mohd Wasif Khan, Daryl Lerh Xing Fung, Robert J. Schroth, Prashen Chelikani, Pingzhao Hu

**Affiliations:** 1 Department of Biochemistry and Medical Genetics, University of Manitoba, Winnipeg, MB, Canada; 2 Children’s Hospital Research Institute of Manitoba, Winnipeg, MB, Canada; 3 Department of Computer Science, University of Manitoba, Winnipeg, MB, Canada; 4 Department of Preventive Dental Science, University of Manitoba, Winnipeg, MB, Canada; 5 Department of Pediatrics and Child Health, University of Manitoba, Winnipeg, MB, Canada; 6 Manitoba Chemosensory Biology Research Group, Department of Oral Biology, University of Manitoba, Winnipeg, MB, Canada; 7 Department of Biochemistry, Western University, London, ON, Canada

**Keywords:** Dentistry, Microbiome

## Abstract

Early childhood caries (ECC) is a multifactorial disease with a microbiome playing a significant role in caries progression. Understanding changes at the microbiome level in ECC is required to develop diagnostic and preventive strategies. In our study, we combined data from small independent cohorts to compare microbiome composition using a unified pipeline and applied a batch correction to avoid the pitfalls of batch effects. Our meta-analysis identified common biomarker species between different studies. We identified the best machine learning method for the classification of ECC versus caries-free samples and compared the performance of this method using a leave-one-dataset-out approach. Our random forest model was found to be generalizable when used in combination with other studies. While our results highlight the potential microbial species involved in ECC and disease classification, we also mentioned the limitations that can serve as a guide for future researchers to design and use appropriate tools for such analyses.

## Highlights

• Early Childhood Caries (ECC) is complex, with the microbiome playing a key role
• Our study combined data from small cohorts to analyze ECC microbiome composition
• We developed a unified pipeline and batch correction to ensure robust analysis
• Our findings suggest key microbial species for ECC and highlight research limitations

## Graphical abstract

**Figure:**
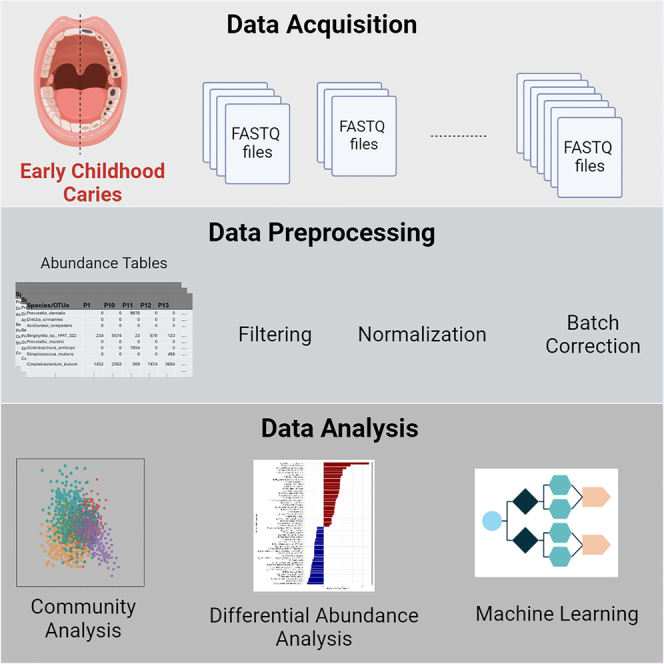

## Introduction

The human body comprises roughly equal numbers of microorganisms as of host cells.^1^^,^^2^ The microorganisms and their genomes found in the oral cavity are collectively referred to as the oral microbiome.^3^^,^^4^ Approximately 800 species have been identified in the human oral microbiome database (HOMD), including both cultivable and not-yet-cultivated species.^5^ Dysbiosis of the microbiome causes several oral diseases, including dental caries. Caries is the term used for tooth decay, and it is the most prevalent infectious disease in the oral cavity. Caries in children less than 72 months involving the primary dentition is known as early childhood caries (ECC) and affects about half of children worldwide.^6^^,^^7^

Dental caries is multifactorial in origin and one of the main drivers for caries development is the biochemical transformations caused by acidogenic microbes on the dental surface.^8^^,^^9^ There is a hypothesis of relatively lower bacterial diversity in carious lesions due to constant acidic exposure.^10^ This change can be observed in the dominance of aciduric microbes. *Streptococcus mutans* is found to be the main caries-causing bacteria, but previous studies have identified several other bacteria involved in caries, such as *Actinomyces* and *Lactobacillus*.^6^^,^^11^^,^^12^ Several acidogenic species have been identified in the microbiome of children with ECC, including *Scardovia wiggsiae*, *Lactobacillus salivarius*, *Streptococcus mutans*, and *Parascardovia denticolens*.^13^

Since the arrival of next-generation sequencing (NGS), amplicon sequencing using the 16S rRNA gene has been the predominant method for identifying and quantifying bacterial communities in complex biological samples.^14^ NGS provides a platform to sequence the culturable or non-culturable strains using short sequence reads to study the oral microbial community. The most common sites to study the oral microbiome are saliva and supragingival plaque, although there are site-based differences in microbiomes especially in the context of ECC.^15^ Furthermore, there is no standard approach yet regarding which region of 16S rRNA has to be used, and this introduces biases among studies and also makes it difficult to resolve it beyond the genus level.^16^

Microbiome biomarkers have been extensively studied in gut microbiomes for colorectal cancer, diabetes, obesity, and inflammatory bowel disease. Given the high dimensionality of microbiome profiles in any study, robust models are required to identify predictive features and ensure the reproducibility of the analysis. One of the goals of supervised classification methods in microbiome analysis is to identify the predictive features for a given condition and produce a predictive model. In addition to the high dimensionality and high sparsity of microbiome data, one of the key challenges is the limited number of common species among samples. Based on the popularity of machine learning (ML) methods, a recent review suggested four common methods for microbiome-based classification analysis: random forest, support vector machines (SVM), logistic regression, and k-nearest neighbor (KNN).^17^

Meta-analysis can help address discrepancies that arise due to technical and/or biological inconsistencies between studies.^18^ It also enables the identification of the universality of the biomarker for specific disease diagnosis or prognosis. On the other hand, it is also important to understand the factors underestimating the effects of these analyses. Different procedures for sample handling, sequencing methods, and data preprocessing methods can lead to discrepancies in the datasets. Hence, it is important to address such issues and minimize these factors to the extent possible. Several studies have applied meta-analysis to various diseases, such as Parkinson’s disease,^19^ cancer,^20^^,^^21^ and urolithiasis.^22^ To our knowledge, no previous study has combined microbiome data from different studies in order to undertake a meta-analysis of the microbiome of children with ECC. Such an analysis would enable us to understand the complexity of microbial profiles and site-specific complexity in ECC.

**Figure 1:**
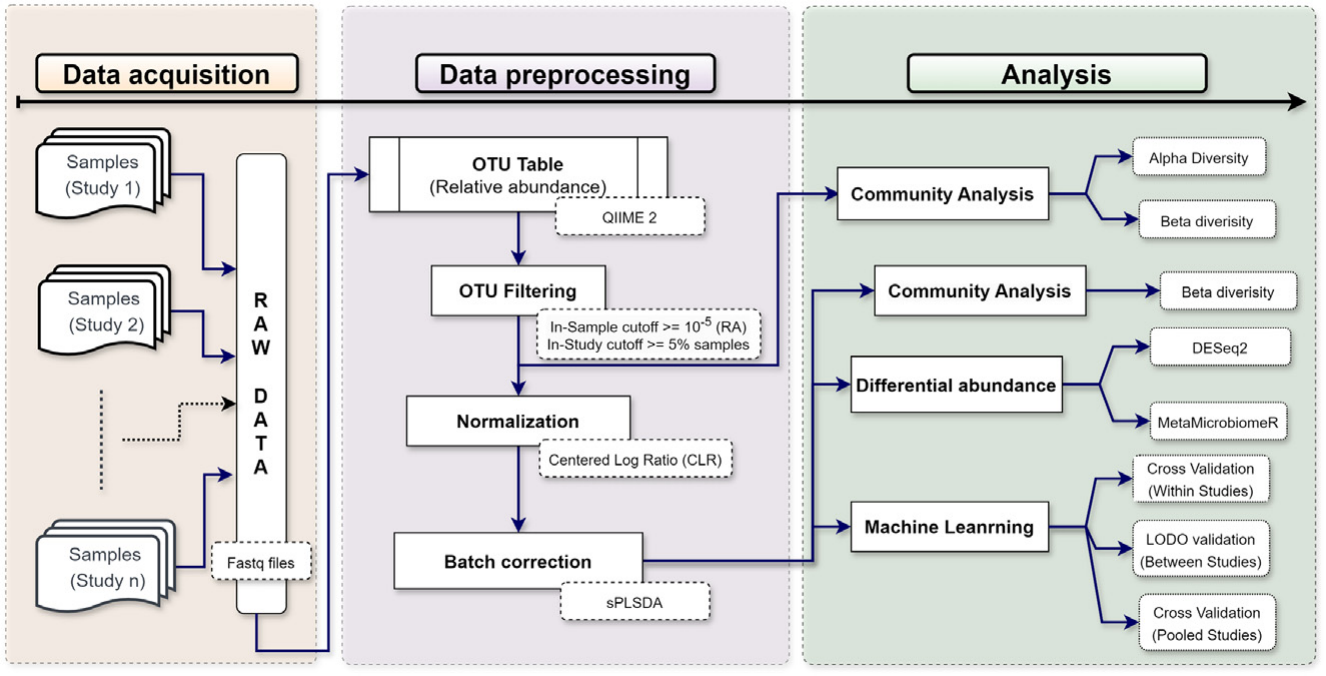
Schematic overview of data processing and analysis pipeline

In this study, we analyzed five published studies on ECC, which made the raw data publicly available based on 16S rRNA amplicon sequencing. We processed the raw data using the same approach wherever possible. This combined analysis helps to consolidate the diverse outcomes observed across individual studies. The meta-analysis pipeline we report in this study provides robust results for the ECC classification and identification of taxonomic biomarkers. Figure 1 illustrates an overview of the overall study design.

## Results

### Datasets

**Figure 2:**
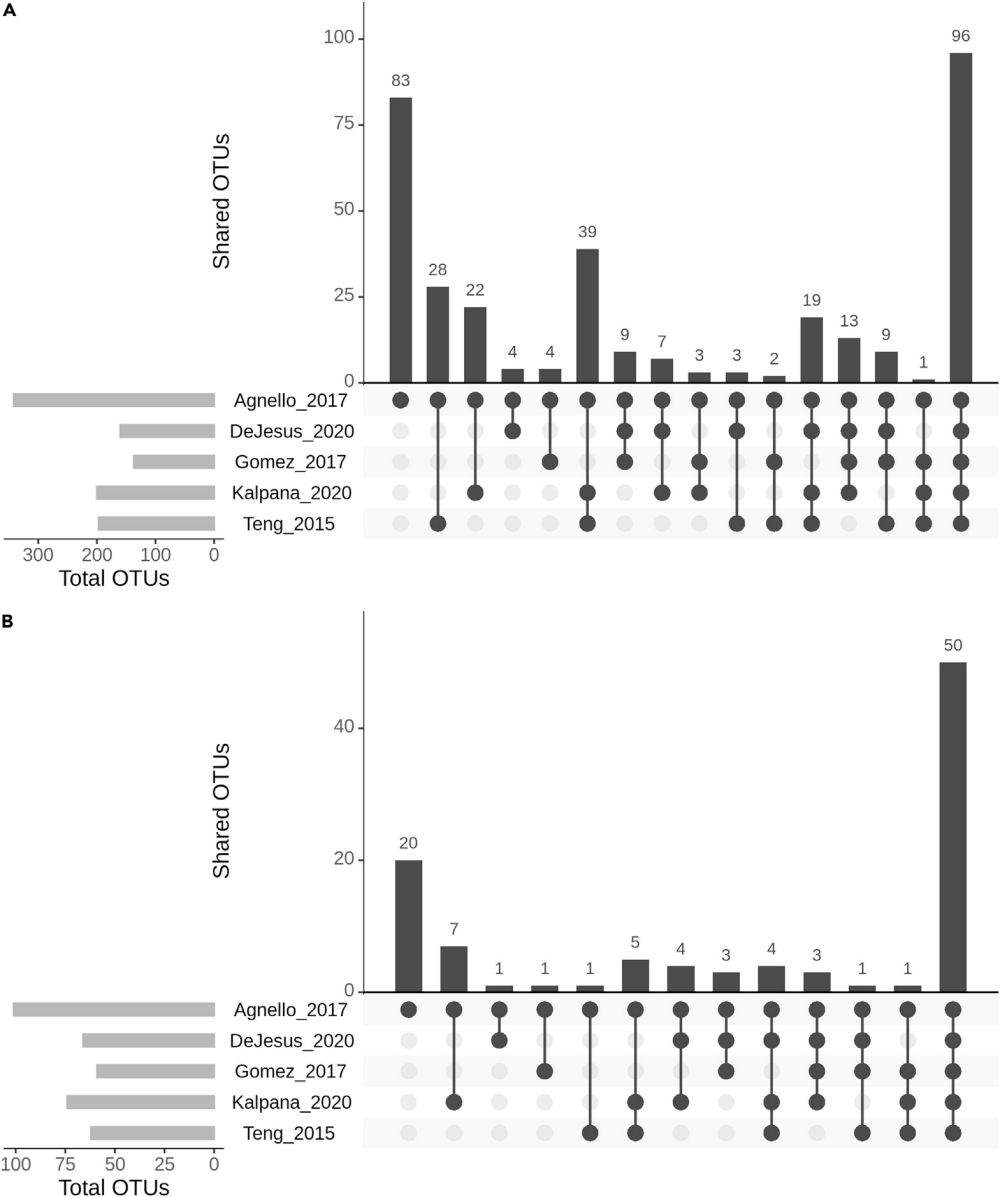
UpSet plot for the number of shared OTUs across different datasets (A) Genus level. (B) Species level. The smaller subplot represents the total number of OTUs at the respective taxonomic level. The main bar plot shows the number of unique and shared OTUs between the studies. The connecting dots below indicate the intersection of the studies indicated by each bar.

Based on our selection criteria, we identified five studies that were included in this analysis.^6^^,^^23^^,^^24^^,^^25^^,^^26^ Data collection from these datasets resulted in a total of 223 samples:117 caries-free (CF) and 106 ECC. The data for all the datasets except Agnello_2017 were downloaded from sequence read archive (SRA) in the form of FASTQ format and the data for Agnello_2017 was obtained from the authors upon request.^23^ The description of the datasets about the sample size, case-control number, additional metadata given about the samples, and accession number for the raw data included in the analysis is given in Table 1. In the operational taxonomic units (OTU) tables of five processed datasets, the genus-level taxa ranged from 59 to 101 with 50 common genera (Figure 2A). While at species level, the total number of OTUs ranged from 133 to 342 with an intersection of 96 species common across all the samples (Figure 2B).

**Table 1:** Information about the datasets used

Study name	Samples^a^		16S rRNA region	SRA accession	Metadata information available	Location of the sample collection
Agnello et al.^23^	20 CF	30 ECC	V3-V4	Obtained from the authors	None	Winnipeg, Canada.
Gomez et al.^24^	20 CF	12 ECC	V4	PRJNA383868	Age, Sex	Adelaide/Melbourne/Sydney, Australia
Kalpana et al.^25^	10 CF	11 ECC	V3-V4	PRJNA454811	None	Tiruchengode, India
Teng et al.^6^	27 CF	13 ECC	V1-V3	SRP040945 and SRP040947	Age, dmfs^b^	Guangzhou, China
DeJesus et al.^26^	40 CF	40 ECC	V4	PRJNA555320	Age, Sex	Winnipeg, Canada

a CF: caries-free; ECC: early childhood caries.

b dmfs: decayed, missing, filled surfaces score.

### Diversity analysis

**Figure 3:**
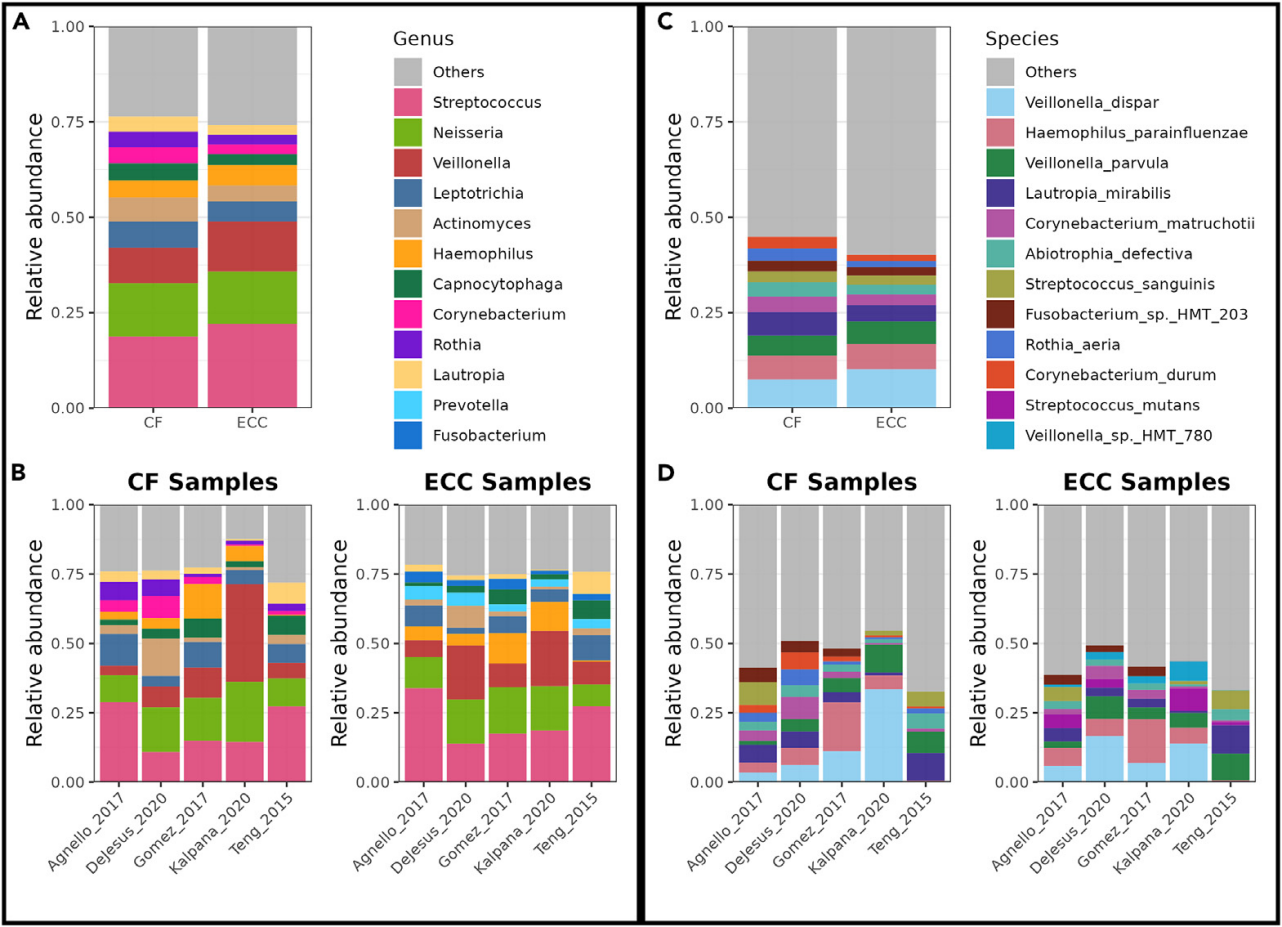
Abundance of top taxa between caries and ECC samples (A) Combined datasets at the genus level. (B) Individual datasets at the genus level. (C) Combined datasets at the species level. (D) Individual datasets at the species level.

**Figure 4:**
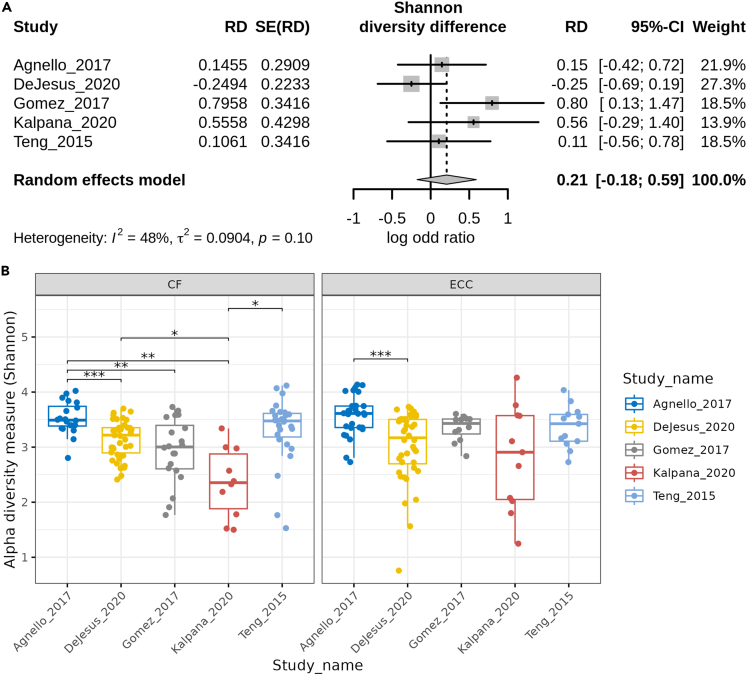
Comparison of species-level alpha diversity by Shannon index (A) The forest plot compares the log odd ratios (with a 95% confidence interval) of the Shannon diversity difference between CF and ECC samples for each dataset. The TE and seTE columns provide the total estimate and standard error of the total estimate values, respectively. (B) Comparison of alpha diversity for CF and ECC samples between datasets using a t-test with significance levels of ∗ = *p* < 0.05, ∗∗ = *p* < 0.01, and ∗∗∗ = *p* < 0.005.

**Figure 5:**
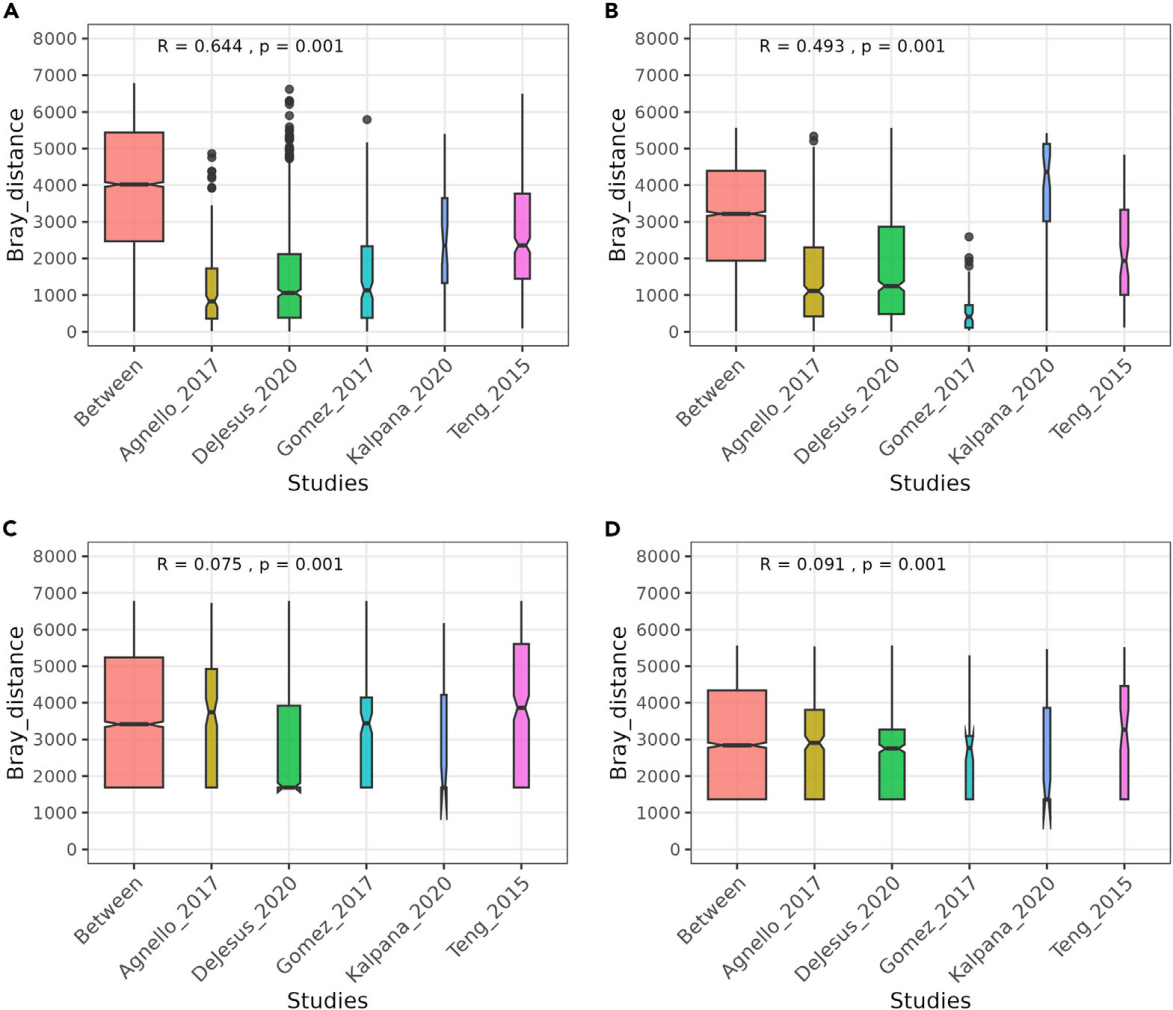
Analysis of similarities (ANOSIM) test using Bray diversity metrics on species-level OTUs to illustrate the extent of similarities between five studies (A) CF group before batch correction. (B) CF group after batch correction. (C) ECC group before batch correction. (D) ECC group after batch correction. An R value close to “1.0” suggests dissimilarity between groups while an R value close to “0” suggests an even distribution of high and low ranks within and between groups.” The *p* values represent the significance of the R value. The first column in each group of boxplots represents the between-group dissimilarity, and the other five columns represent the beta diversity within each study. The figure shows that there was a significant drop in between-study dissimilarity after the sPLSDA batch correction method. The size of the box is proportional to the sample size in each dataset.

**Figure 6:**
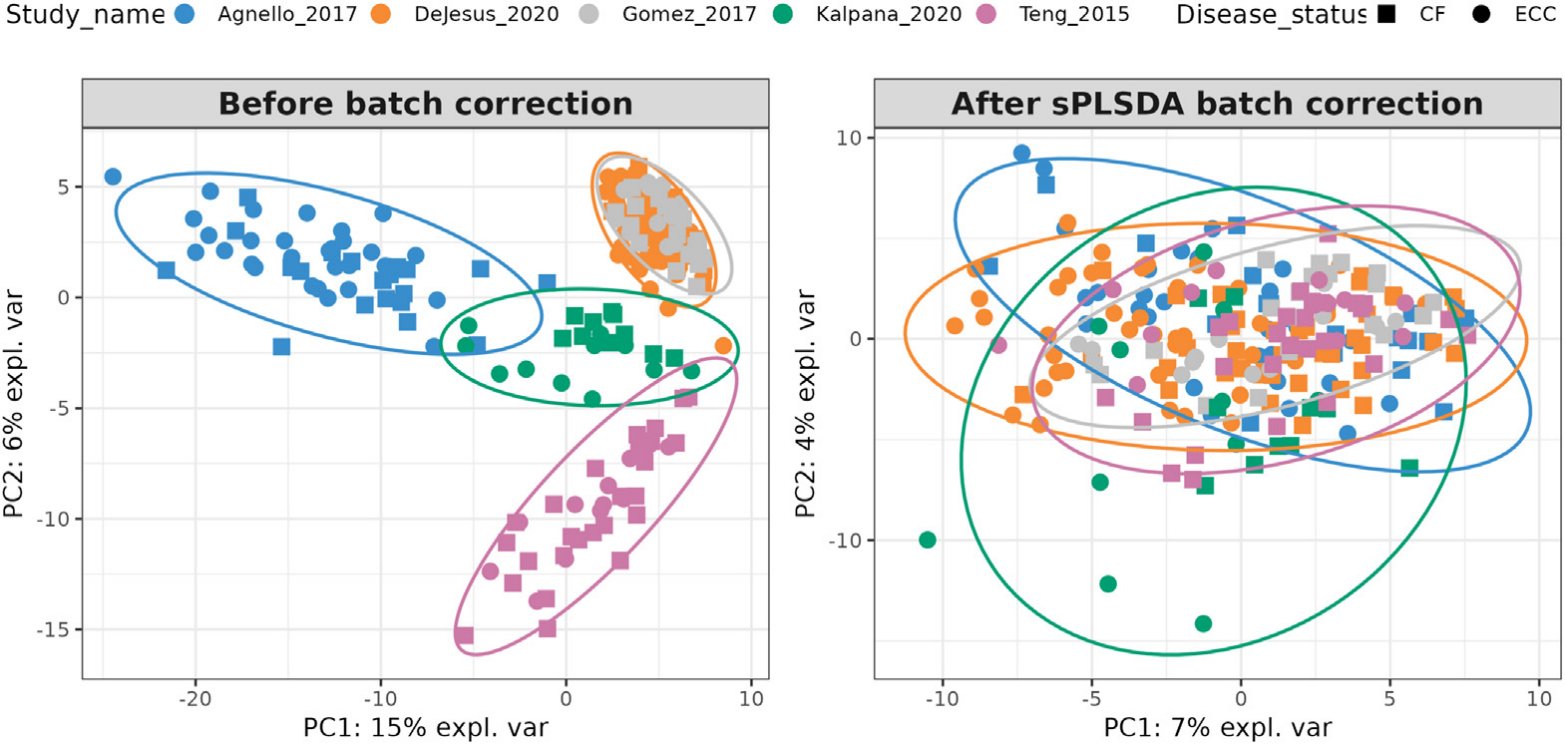
PCA plots for species-level datasets (A) Using relative abundance data before any batch correction. (B) After sPLSDA batch correction on centered log ratio (CLR) transformed values. The PC1 and PC2 axes also include the explained variance of these PC components. The batch correction can be noticed by the reduction in explained variance on the PC1 and PC2 axes.

The most common genera in both CF and ECC samples were *Streptococcus*, *Neisseria*, and *Veillonella* (Figure 3). Among all species, *Veillonella* species were abundant. The meta-analysis for Shannon diversity at the species level revealed that the diversity for disease status did not significantly change between CF and ECC samples, and the heterogeneity for diversity was found to be 48 percent (Figure 4). However, a significant change among the CF samples between the studies can be observed. While this is not the case among ECC samples, a significant diversity change is only observed between Teng_2015 and Kalpana_2020 datasets. For the species-level beta diversity analysis, the analysis of similarities (ANOSIM) statistic R value was 0.64 and 0.49 for CF and ECC groups, respectively (Figure 5). A reduction in R values was observed after batch correction (CF = 0.075 and ECC = 0.091), which is suggestive of a decrease in dissimilarity or increased homogeneity in beta diversity (Figures 5 and 6).

### Differential abundance analysis

**Figure 7:**
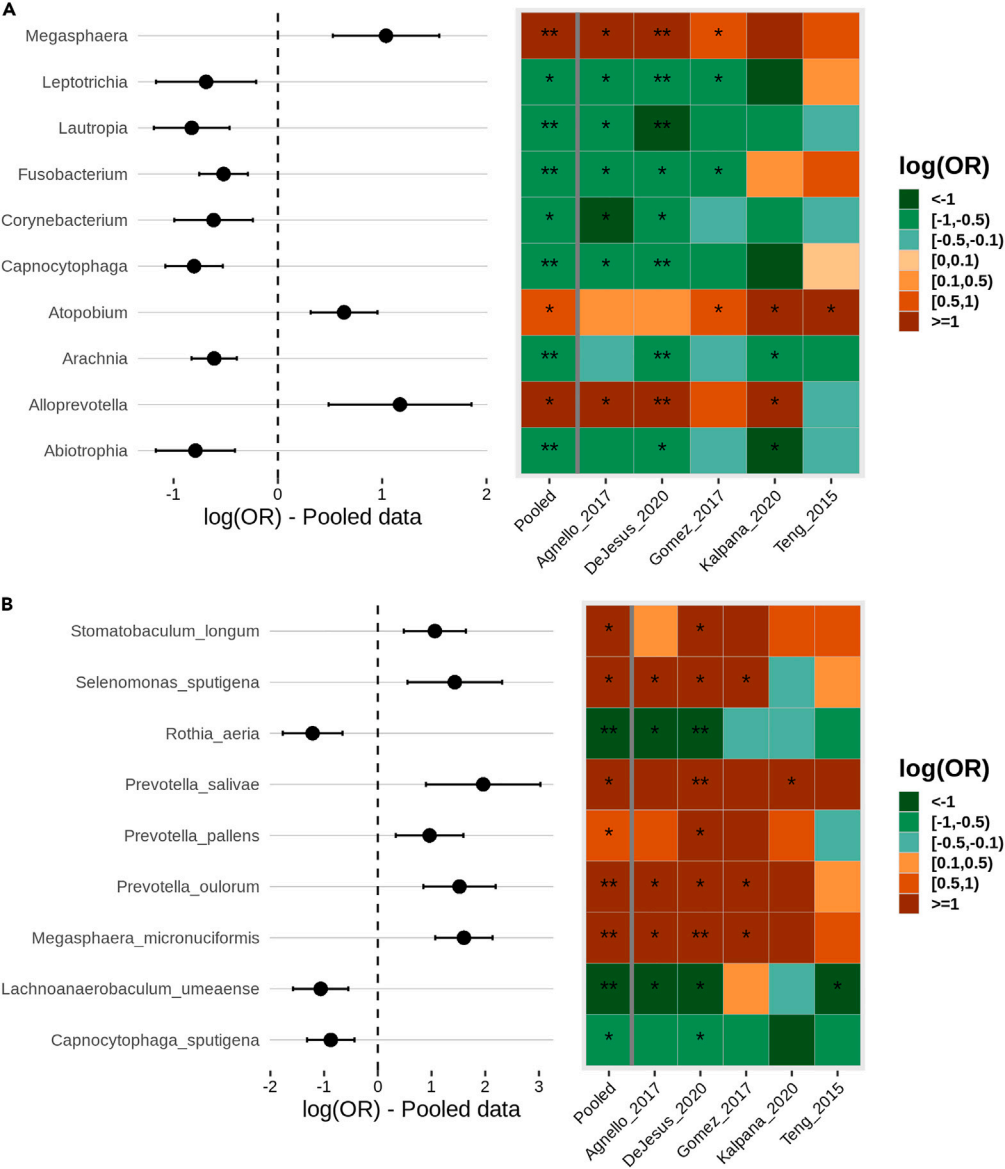
Meta-analysis for differentially abundant taxa (A) Genus level. (B) Species level. The heatmap represents the log odd ratios of differentially abundant with significant p-adjusted value (p-adjusted<0.05) in pooled dataset along with the odd ratio estimates in each dataset. The forest plot signifies a 95% confidence interval for the log odd ratio values for each taxon in the pooled dataset.

For differential abundance analysis, Datasets DeJesus_2020, Agnello_2017, and Kalpana_2020 resulted in a higher number of differentially abundant taxa than the remaining two datasets at both species and genus levels. In our analysis, we found that DESeq2 identified more species as differentially abundant than the LefSe method. The species identified by DESeq2 methods in all the datasets are illustrated in Figure S1. The meta-analysis of differentially abundant species revealed a higher abundance of *Prevotella salivae*, *Selenomonas sputigena, and Prevotella oulorum* in the ECC group, while at the genus level, *Alloprevotella* and *Megasphaera* were found to be associated with ECC (Figure 7). A comparison of differential abundance taxa for relative abundance and batch-corrected data suggests that the significantly altered OTUs are more consistent with batch-corrected values, and the number of such OTUs is higher for adjusted *p*-values. The batch-corrected data provided nine significant species in comparison with two species with relative abundance data (Figures 7 and S2).

### Classification of CF and early childhood caries using machine learning

**Figure 8:**
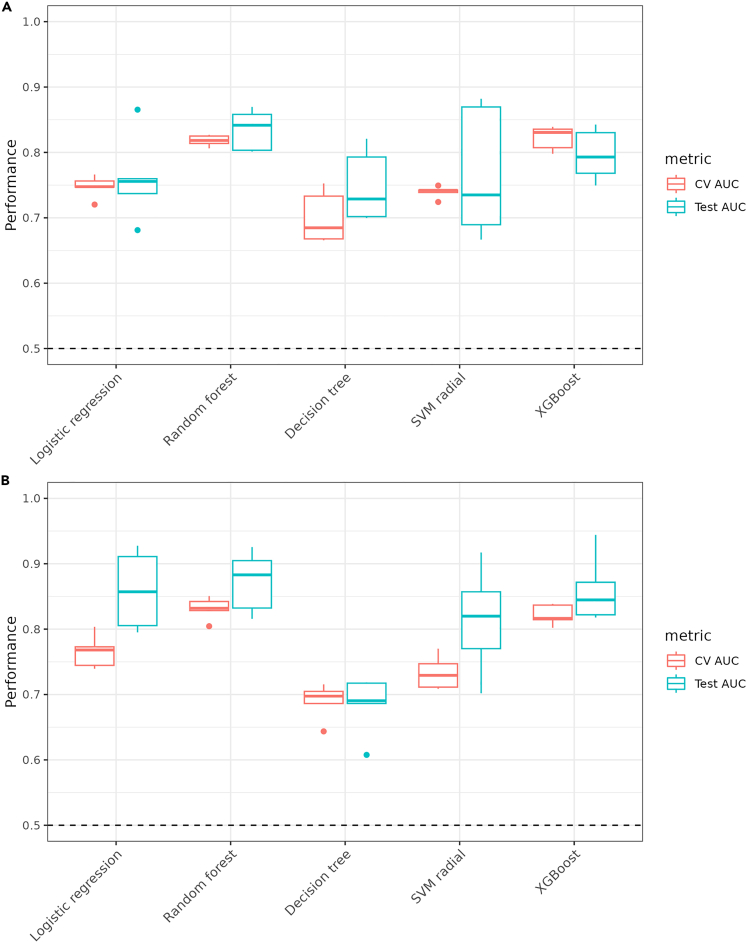
Model performance by the area under a receiver operating characteristic curve for 5 different machine learning models on the pooled dataset from 5 studies (A) Genus level. (B) Species level. The x axis represents the models used for the comparison of the performance. CV-AUC and test-AUC boxes denote the AUROC values from cross-validation and test data, respectively, from pooled datasets. These results indicate that the average performance of the random forest method outperforms the other methods compared here.

**Figure 9:**
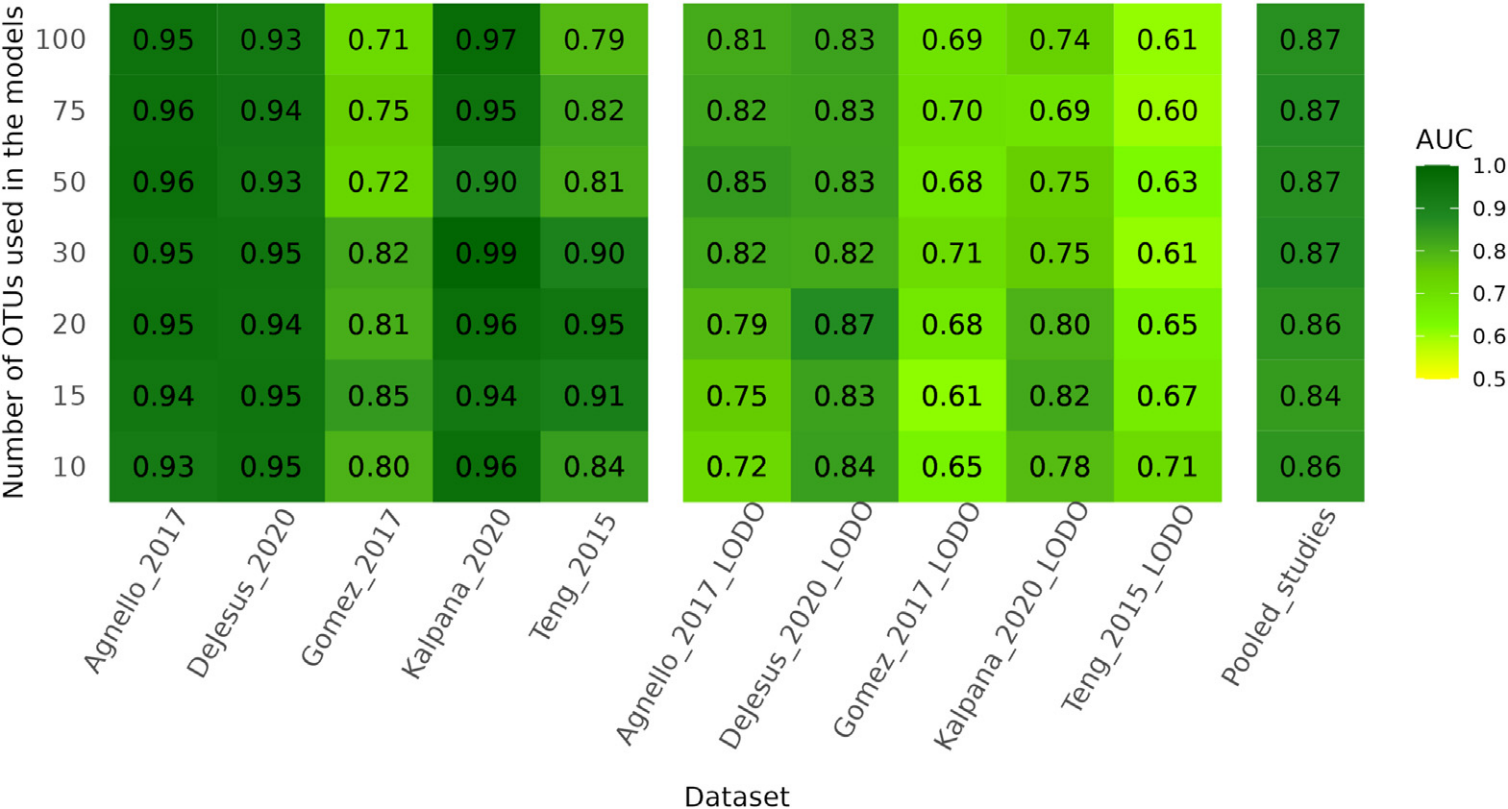
Heatmap for the performance of random forest classifier by Area under ROC curve (AUROC) on species-level data The left panel shows the cross-validation results for each dataset. The middle panel shows the model performance for LODO analysis. In the LODO analysis, all datasets except one were used for training, and the left-out dataset was then used for testing to assess the generalizability of the model. The rightmost column shows the cross-validation performance of the pooled dataset. The y axis represents the number of top OTUs used for model assessment.

**Figure 10:**
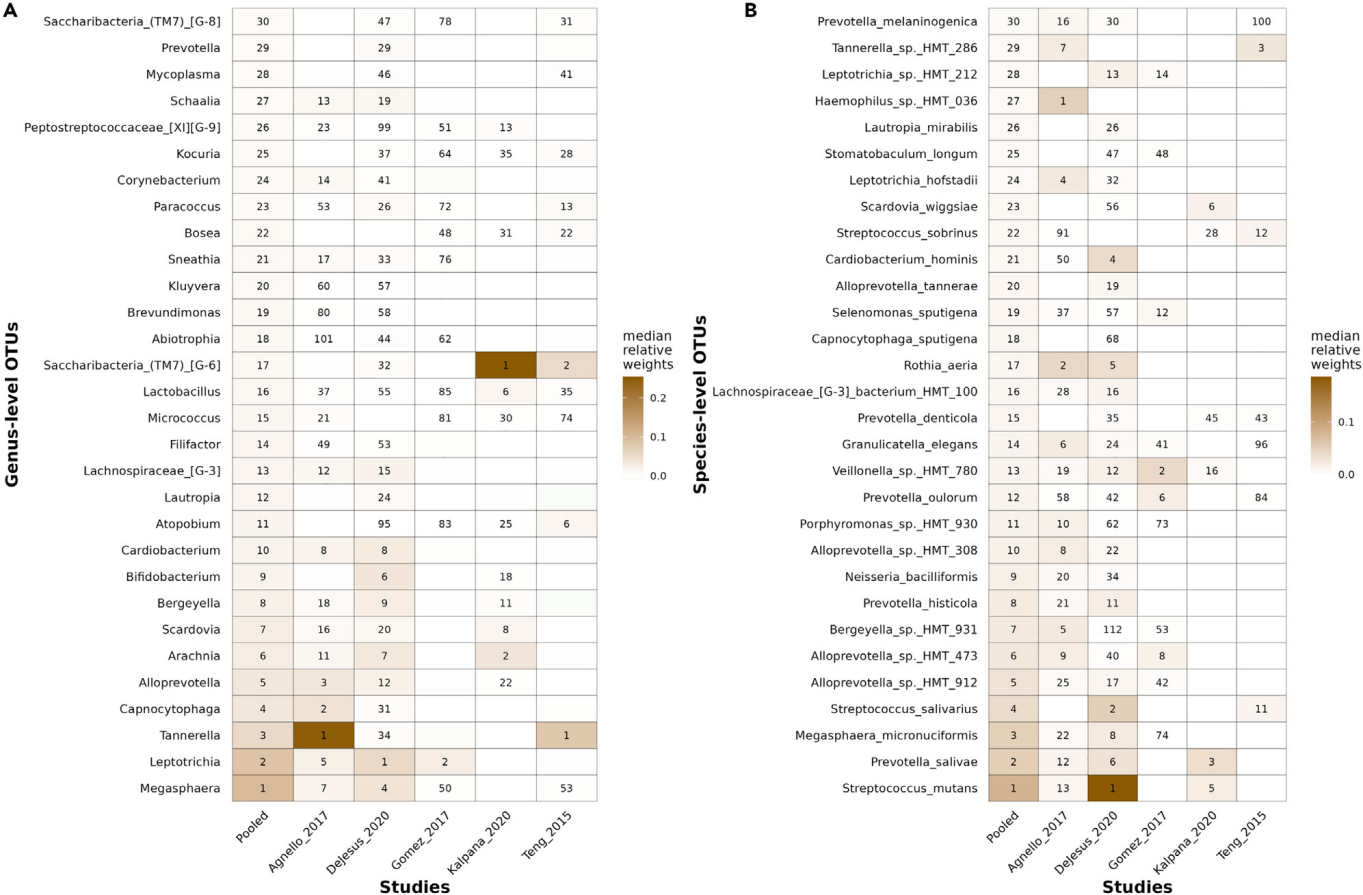
Heatmap for feature relative weights along with their rank obtained from the random forest model (A) Genus-level OTUs and (B) Species-level OTUs. The top 30 features in the pooled dataset were used to compare the feature weights from the individual datasets. The random forest model was used for each dataset with a 5-fold cross validation and five repeats. The weights were obtained by the median of the feature weights from each model.

For the classification of CF and ECC samples using machine learning methods, we tested some commonly used ML methods in metagenomic studies, which are logistic regression with Lasso penalty, random forest, XGBoost, SVM, and decision trees. The performance for cross-validation and test set validation was the best with random forest and XGBoost, while lasso results were very close to these two methods (Figure 8B). The average classification performance in terms of area under the receiver operating characteristic curve (AUROC) value is 0.85 at the species level and 0.83 at the genus level using random forest. When the OTUs for both genus and species levels were combined, the AUROC value was 0.84 (Figure S3). The performance of random forest was best with 20–40 OTUs selected from random forest models based on feature weights, and it did not change significantly with a higher number of OTUs (Figure 9). Here, we used the term within-study-cross-validation (CV) for the performance within the same study, and leave-one-dataset-out (LODO) analysis when applying a model trained on one study and tested on another study. The random forest method outperformed the other ML methods used in this study for LODO analysis as well. We also explored the importance of the features obtained from the models for individual studies and pooled datasets (Figure 10). Comparatively, genus-level OTUs were more common among studies than species-level OTUs. At the species level, Agnello_2017 and DeJesus_2020 shared many OTUs used in model development.

## Discussion

Small and heterogeneous cohorts provide distinct opportunities to study disease-specific microbiomes.^21^ We extended this idea to study the ECC-associated microbiome by combining them to achieve a better representation of ECC cases and controls. In our analysis, we combined previously published dental plaque microbiome datasets for ECC children and analyzed them using a common data processing pipeline to understand the patterns across cohorts and test the generalizability of machine learning classifiers across datasets.

A general practice in microbiome data analysis is to transform read counts to relative abundance or to rarify the data to a certain number of reads. However, significant variation can be observed among the studies in terms of OTUs, even after such transformations. These differences can arise from discrepancies in sample handling, DNA extraction methods, and sequencing techniques. To minimize these batch effects, we first processed the raw reads using a common pipeline whenever possible. Second, we used the sparse Partial Least Squares Discriminant Analysis (sPLSDA) batch correction method, which has shown better efficiency in reducing batch effects in microbiome data than other batch correction methods.^27^ sPLSDA methods capture more variance due to the treatment than the commonly used methods such as ComBat.^28^ For compositional data, the CLR method addresses the variability in library sizes and diminishes the skewness present in the data.^29^ Moreover, CLR transformation approximates a Gaussian distribution on the count data which provides a better performance using the sPLSDA method.^28^

We identified very few significant species in the differential abundance analysis in some of the datasets. One reason could be the power of the statistical test – studies with more samples resulted in a higher number of significant taxa, for example, the DeJesus_2020 dataset (Table 1). Another reason for that could be the limited number of total reads in certain samples. For optimal taxonomic coverage, it is recommended to have approximately 5000 reads per sample. Although most of the samples in our study had more than the specified number of reads, sometimes samples with a smaller number of reads, as low as 3000, were also included due to the small sample size in some of the datasets.

Commonly used machine learning methods in microbiome studies include logistic regression models, SVM, random forest, and gradient boosting methods, such as XGBoost. In our analysis, the random forest method outperformed the other tested methods in both cross-validation and the train-test strategy when applied to the pooled dataset (Figure 8). The observation of lower CV-AUROC values in these results can be attributed to the diversity within each fold which will lead to the poorer average performance of CV-AUROC. However, the final model may have leveraged from the combined coefficients of all folds which captures more nuances and generalizes better on the test dataset. Although comparable with relative abundance, the batch-corrected data give more consistent results with random forest, and the performance linearly improves with the number of features. However, it did not change significantly after 20–30 features in terms of OTUs (Figure 9). Previous studies have also shown that random forest provides better classification than other commonly used ML methods in the meta-analysis of microbiome data.^21^^,^^30^

Our meta-analysis and machine learning analysis revealed some important taxa identified in this cross-cohort analysis. *Streptococcus* species (for example, *S. mutans*, *S. salivarius*, *S. oralis,* and S. sp. HMT-074) were dominant in the top species from the machine learning models. Interestingly, *Streptococcus* species were not identified in the meta-analysis and genus-level machine learning analysis. The role of *Streptococcus* species has been highlighted in previous studies; for example, *S. mutans* and *S. oralis* were discussed as high-risk factors in ECC.^6^^,^^31^ *Streptococcus* species ferment sugars to produce lactic acid, which reduces the pH and causes the demineralization of enamel.^32^ A novel host-microbe interaction between the cariogenic *S.mutans* and taste receptors on the oral gingiva leading to innate immune responses was reported.^33^ It was suggested that *S.mutans* might be modulating the immune system to inhibit other gram-positive bacteria and involved in mediating autophagy flux in gingival epithelial cells.^34^^,^^35^ The next most significant genera were *Prevotella* and *Alloprevotella*. The species from these genera were significant in both meta-analysis and machine-learning analysis at the species level. *Prevotella* and *Alloprevotella* are genera enriched in increased dental caries.^36^^,^^37^ *Alloprevotella* species were found to be mild saccharolytic and acid-producing as a result of fermentation.^37^ Another genus, *Tannerella*, was also found to be important from ML-based feature analysis at both the species and genus levels.

To illustrate that the classifier performance with batch correction data is largely due to the batch correction method and not the underlying CLR normalization, we also compared the AUROC values for CLR-transformed data and batch-corrected values (Figures 9 and S4). From these results, it is evident that although CLR improves cross-validation within studies, the LODO performance of the CLR method alone is poorer than the machine learning performance on sPLSDA batch-corrected data.

Our results demonstrate that LODO analysis provides generalizability for the classification of CF and ECC using dental plaque microbiome data. Results for LODO analysis are very promising, reaching as high as 0.88 in terms of AUROC values for the classification of ECC and CF status. Another conclusion is that species-level analysis provides better results than genus-level analysis (Figures 9 and S5). We also compared the features identified in each study for within-study-CV at the genus level for plaque samples and observed that there were very few common features among studies, and no single feature was common across all five datasets (Figure 10).

These results also suggest that the classification performance and differentially abundant species could be more robust with larger datasets, as observed in the DeJesus_2020 and Agnello_2017 datasets. Additionally, microbiome-based diagnostic biomarkers can help in the early prediction of severe ECC, which can help clinicians and parents to adopt timely preventive measures. It can help improve the quality of life at both the individual and societal levels. Future prospective studies are needed to establish a causal link between these biomarkers and ECC.

Machine learning classifiers work better when the dataset is the union of all studies for OTUs instead of only common OTUs (data not shown). This is potentially due to the loss of a substantial number of features when considering the intersection of the datasets for OTUs, which reduced to 96 from 391. However, the union of datasets induced 0 inflation for the OTUs that were not present in the original OTU table. To circumvent this problem, several ML methods have relied on imputation techniques. In microbiome data analysis, the only method available specifically for microbiome data is mbImpute. We included the results of random forest performance imputing the missing values using the mbImpute method (Figure S6). However, we did not observe any improvement in the classifier’s performance. Further exploration using imputation methods is required in microbiome studies.

In summary, we conducted a meta-analysis of dental plaque ECC-associated microbiomes from five previously published studies. We assessed the importance of normalization and batch correction methods when conducting such analyses. Our meta-analysis included the differential abundance and machine learning-based identification of features in combined studies. Based on high value AUROC value in LODO analysis, we conclude that the ECC microbiome shows a good extent of common features across different studies worldwide, which can be studied with appropriate data merging techniques and modern machine learning tools.

### Limitations of the study

Some possible limitations of our analysis could be as follows: the number of reads varied greatly between samples, and each study used a particular variable region or combination of the variable regions of 16S amplicon sequencing to identify the bacterial community.^38^ The differences in the datasets might be due to the different regions selected during sequencing, as shown in Table 1.^38^ Another potential variance in the caries microbiome may stem from different areas of the teeth. These differences have been observed between occlusal and proximal caries, as well as between enamel and dentin caries in ECC.^13^^,^^39^ However, the samples used in our analysis were not collected from specific tooth surfaces, which may limit the applicability of our findings to the differences arising from distinct sites.

## STAR★Methods

### Key resources table

**Table:** 

REAGENT or RESOURCE	SOURCE	IDENTIFIER
**Software and algorithms**		

fasterq-dump v 2.9.6	NCBI SRA-toolkit	https://trace.ncbi.nlm.nih.gov/Traces/sra/sra.cgi?view=software
QIIME2 v 2021.2	Bolyen et al.^40^	https://docs.qiime2.org/2021.2/
HOMD v 15.22	Escapa et al.^5^	https://v2.homd.org/
mixOmics v 6.20.0	Rohart et al.^41^	https://bioconductor.org/packages/mixOmics/
mbImpute v 0.1.0	Jiang et al.^42^	https://github.com/ruochenj/mbImpute
DECIPHER v 2.24.0	Wright^43^	https://bioconductor.org/packages/DECIPHER/
Phyloseq v 1.40.0	McMurdie and Holmes^44^	https://joey711-github-io.uml.idm.oclc.org/phyloseq/
metamicrobiomeR v 1.2	Ho et al.^45^	https://github.com/nhanhocu/metamicrobiomeR
Vegan v 2.6-4	Dixon^46^	https://github.com/vegandevs/vegan
microbiomeMarker v 1.4.0	Cao et al.^47^	https://bioconductor.org/packages/microbiomeMarker/
UpSetR v 1.4.0	Conway et al.^48^	https://github.com/hms-dbmi/UpSetR/
Mikropml v 1.4.0	Topçuoğlu et al.^49^	https://github.com/SchlossLab/mikropml
SIAMCAT v 2.0.1	Wirbel et al.^50^	https://github.com/zellerlab/siamcat

**Deposited data**		

The source code used in this article	This paper	https://github.com/wasifmohdkhan/ECC_Microbiome_MetaAnalysis_DentalPlauqe

### Resource availability

#### Lead contact

Further information should be directed to Dr. Pingzhao Hu (phu49@uwo.ca).

#### Materials availability

This study did not generate new unique reagents.

#### Data and code availability

• Data: The source of datasets used for analysis during the current study are given in Table 1. Four out of five datasets are publicly available from their respective NCBI SRA repositories. The fifth dataset, not available on NCBI SRA, was obtained from the authors upon request.
• Code: The source code of this article can be publicly accessed at https://github.com/wasifmohdkhan/ECC_Microbiome_MetaAnalysis_DentalPlauqe.
• Any additional information will be made available upon request from the lead contact.

### Experimental model and study participant details

This meta-analysis is based on previously published studies. Participant details can be found in the articles related to their respective studies (Table 1). Inclusion and exclusion criteria used in this study are provided in the sections below and in Table S1.

### Method details

#### Inclusion and exclusion of studies

Studies were selected from publications that conducted microbiome comparisons between caries-free (CF) individuals and those with early childhood caries (ECC) using a case-control design. Among these studies, only those employing 16S rRNA amplicon sequencing and providing the raw sequencing data were selected. The raw data in such studies can be obtained from one of two techniques:16S rRNA amplicon sequencing and shotgun metagenomic sequencing. Generally, amplicon sequencing using 16S rRNA sequences can be obtained using one or a combination of more than one hypervariable region of the 16S rRNA gene or with full-length 16S rRNA sequencing. For our analysis, studies with 16S rRNA amplicon sequencing data, irrespective of the hypervariable region, were selected. Studies that utilized shotgun metagenomic sequencing methods were excluded from our analysis to minimize the known technical effects. The two most common oral sites for amplicon sequencing are the supragingival plaque (also referred to as dental plaque) and saliva. To avoid site-based differences, we focused on studies that collected their samples from dental plaque.

#### Collection of data and raw data processing

The data were downloaded from NCBI repositories using Fasterq-dump (NCBI SRA-toolkit). Some of the raw sequences were obtained directly from the authors upon request (Table 1). Raw FASTQ sequences were analyzed using the QIIME2 pipeline to obtain the operational taxonomic units (OTUs) table for each study. For the datasets in each study, several combinations of left and right trims were optimized using the DADA2 plugin in QIIME2 to obtain the maximum number of non-chimeric reads. The performance of DADA2 trimming was manually assessed for forward and reverse read trimming. The combination that retained the maximum number of non-chimeric reads in the samples, particularly samples with a low number of total reads, was used for final trimming. To classify the amplicon sequencing variants (ASVs) obtained from DADA2 into OTUs, HOMD 15.22 was used as a reference database. The sequences from the reference database were extracted using the primer sequences cited in each study’s original publication (Table S1). The final OTU tables were obtained at both the genus and species levels. All the tools used to process the datasets can be found in the key resources table and Table S2.

#### Preprocessing

Samples with fewer than 3000 amplicon sequencing variants (ASVs) after the DADA2 step were excluded from all downstream analyses. The raw read counts in all samples were scaled with total sum scaling relative abundance. Further filtering was applied for the OTUs present in less than 5 percent of the samples or OTUs with a maximum value of 10^−5^ relative abundance within a study. Since the samples in our analyses came from different studies, we performed batch correction between studies for our analysis. For batch correction, the relative abundance values were first normalized using centered log-ratio (CLR) normalization and batch correction was performed using the sPLSDA method from the mixOmics package in R.^27^ To test the role of imputation on the classification performance, the mbImpute method was used.^42^ For the imputation step, the phylogenetic distance between OTUs was calculated from 16S sequences using with ‘Jukes-Cantor’ distance in DECIPHER package.^43^

### Quantification and statistical analysis

#### Diversity analysis

We compared the alpha and beta diversities of species-level OTUs using relative abundance. To explore the change in diversity for caries status, alpha diversity with the Shannon index was estimated using the R-package Phyloseq. A meta-analysis was conducted on Shannon diversity changes for individual and pooled studies, and alpha diversity heterogeneity was assessed for these datasets. Shannon diversity differences within samples of CF and ECC children across the datasets were also examined. The significance of the difference between the groups was calculated by t-test, and *p*-values were adjusted by the “holm” method in the R-package rstatix. For beta diversity comparison, owing to the different group sizes, we used analysis of similarities (ANOSIM) with the Bray distance method and compared the beta diversity before and after batch correction.

#### Differential abundance analysis

For differential abundance analysis, two very commonly used methods, LefSe and DESeq2, were applied with multiple hypotheses testing corrections using the false discovery rate (FDR) with adjusted-p < 0.05, using the R package microbiomeMarker.^47^ UpSet plots were used to illustrate the shared and unique differentially abundant species between studies. Furthermore, for the meta-analysis of differentially abundant taxa, we used metamicrobiomeR in R.^45^ For the meta-analysis of differentially abundant species, we used two approaches: GAMLSS-BEZI (Generalized Additive Models for Location Scale and Shape with beta zero-inflated family) on relative abundance and LM (linear mixed effect models) approach on batch-corrected data from the R package metamicrobiomeR.

#### Machine learning modeling

For the classification of CF and ECC samples, logistic regression with the lasso penalty, random forest, decision trees, extreme gradient boosting (XGBoost), and SVM methods were used. For machine learning, 5-fold cross-validation (CV) with five repeats was used. The leave-one-dataset-out (LODO) strategy was used for machine learning-based cross-cohort analysis. In the LODO analysis, all datasets, except one, were used for training with CV, and the left-out dataset was then used for testing to assess the generalizability of the model. The model performance was assessed by area under the receiver operating characteristic curve (AUROC). We extracted the OTU importance for each model in the form of feature weights obtained from the model. The model performance was also compared to the machine learning classifier performance for relative abundance data, batch-corrected data without imputation, and with imputation.

#### Machine learning hyperparameter tuning

The ML models were used from the mikroml pipeline, which used the caret package from R.^49^ For the training of random forest for LODO analysis and holdout dataset testing, the SIAMCAT package was used.^50^ The logistic regression model was used with a lambda value of 10^−4^ to 10 and alpha = 1 for the lasso penalty. The number of trees in random forest was tried between 100 and 1000 with mtry = c(round(sqrt.mdim/2), round(sqrt.mdim), round(sqrt.mdim ∗ 2)), where mtry and sqrt.mdim are the square root of number of random variables in each tree and the total number of features, respectively. In XGBoost, the learning rate (eta), the maximum depth of a tree (max_depth), and the fraction of samples used to train each boosting round (subsample) were evaluated for which a specific range of values. For the SVM models, the cost parameter (C) and the Gaussian kernel’s standard deviation (sigma) were tuned.
