# Supplementary material for: A cross-cohort analysis of dental plaque microbiome in early childhood caries
Source: iScience. 2024 Jul 4;27(8):110447. doi: 10.1016/j.isci.2024.110447 (PMC11298647; doi:10.1016/j.isci.2024.110447)
Supplement: Document S1. Figures S1–S6 and Tables S1 and S2 [file mmc1.pdf]

iScience, Volume *27*

Supplemental information

A cross-cohort analysis of dental plaque microbiome in early childhood caries

Mohd Wasif Khan, Daryl Lerh Xing Fung, Robert J. Schroth, Prashen Chelikani, and Pingzhao Hu

Supplemental information

Supplementary Tables and Figures

Table S1: **Additional details of datasets and Qiime2 processing options, related to Table 1**. Differences in Qiime2 processing options used for dereplication of raw sequences and primer sequences to extract reference sequences for classifier generation. Primer sequences were taken from the respective papers. Dereplication options were optimized to maximize the number of non-chimeric reads.

**Study**	**Dereplication option and Primer sequences used in Qiime2**	**Additional information about the datasets**
**Agnello_2017**	vsearch cluster-features-de-novo<br>• perc-identity 0.99<br><br>f-primer CCTACGGGNGGCWGCAG<br>r-primer GACTACHVGGGTATCTAATCC	The authors provided the FASTA files and not the FASTQ files. Since the dada2 option cannot be used with FASTA file, we used the vsearch option in Qiime2 with 99 percent similarity for the binning of similar reads.
**DeJesus_2020**	dada2 denoise-paired<br>• p-trunc-len-f 200<br>• p-trunc-len-r 150<br>• p-trim-left-f 15<br>• p-trim-left-r 15<br><br>f-primer GTGCCAGCMGCCGCGGTAA<br>r-primer GGACTACHVGGGTWTCTAAT	
**Gomez_2017**	dada2 denoise-paired<br>• p-trunc-len-f 200<br>• p-trunc-len-r 150<br>• p-trim-left-f 15<br>• p-trim-left-r 15<br><br>f-primer GTGCCAGCMGCCGCGGTAA<br>r-primer GGACTACHVGGGTWTCTAAT	This dataset was composed of participants of 5 to 11 years old. Since the age restriction to be considered as ECC is less than 6 years old children only, participants with age less than 6 years were selected from this database which were 20 Caries free and 12 with caries.
**Kalpana_2020**	dada2 denoise-paired<br>• p-trunc-len-f 250<br>• p-trunc-len-r 230<br>• p-trim-left-f 5<br>• p-trim-left-r 5<br><br>f-primer CCTACGGGNBGCASCAG<br>r-primer GACTACNVGGGTATCTAATCC	The NCBI repository had raw FASTQ reads for 10 caries-free and 11 ECC participants only. These numbers were also confirmed with the authors, and they mentioned that only good-quality samples were deposited in the NCBI SRA repository.
**Teng_2015**	dada2 denoise-pyro<br>• trim-left 20<br>• trunc-len 490<br>• max-ee 5	For this longitudinal study, the authors divided the children into three groups: H2H- Children who remained healthy for the entire duration, H2C- Children who were caries-free at the time of recruitment

	f-primer GAGTTTGATCCTGGCTCAG<br>r-primer TACCGCGGCTGCTGGCAC	but developed caries later, C2C- Children who had caries at the time of recruitment and continued to have caries for the duration of this study. To mimic the samples for case-control type data, we selected all samples from H2H at different data points with confident health (27 samples) and from caries only the samples with dmfs value greater than or equal to 5 were selected which can be considered as severe ECC (SECC). A further sample filtering was done for the samples that retained less than 3000 non-chimeric reads after the dada2 step in Qiime2 processing.

Table S2: Key resources table with additional description, related to Key Resource Table in STAR methods.

RESOURCE	IDENTIFIER	DESCRIPTION
fasterq-dump v 2.9.6	https://trace.ncbi.nlm.nih.gov/Traces/sra/sra.cgi?view=software	Converts SRA data format to FASTQ format.
QIIME2 v 2021.2	https://docs.qiime2.org/2021.2/	Microbial analysis from raw FASTQ sequences to OTU/ASV table.
HOMD v 15.22	https://v2.homd.org/	Human Oral Microbiome Database for curated information on bacteria in the human mouth.
mixOmics v 6.20.0	https://bioconductor.org/packages/mixOmics/	Multivariate analysis of omics biological studies.
mbImpute v 0.1.0	https://github.com/ruochenj/mbImpute	Missing data imputation for microbiome analysis.
DECIPHER v 2.24.0	https://bioconductor.org/packages/DECIPHER/	A toolset for deciphering and managing biological sequences.
Phyloseq v 1.40.0	https://joey711-github-io.uml.idm.oclc.org/phyloseq/	A tool to import, store, analyze, and graphically display microbiome data.
metamicrobiomeR v 1.2	https://github.com/nhanhocu/metamicrobiomeR	R package for analyses and meta-analyses of other microbiome studies.
Vegan v 2.6-4	https://github.com/vegandevs/vegan	This package provides tools for descriptive community ecology.
microbiome Marker v 1.4.0	https://bioconductor.org/packages/microbiomeMarker/	R package for microbiome marker identification and visualization.
UpSetR v 1.4.0	https://github.com/hms-dbmi/UpSetR/	Visualization of complex intersections of datasets.
Mikropml v 1.4.0	https://github.com/SchlossLab/mikropml	Machine learning pipeline for microbiome data.
SIAMCAT v 2.0.1	https://github.com/zellerlab/siamcat	Statistical inference of associations between microbial communities and host phenotypes.

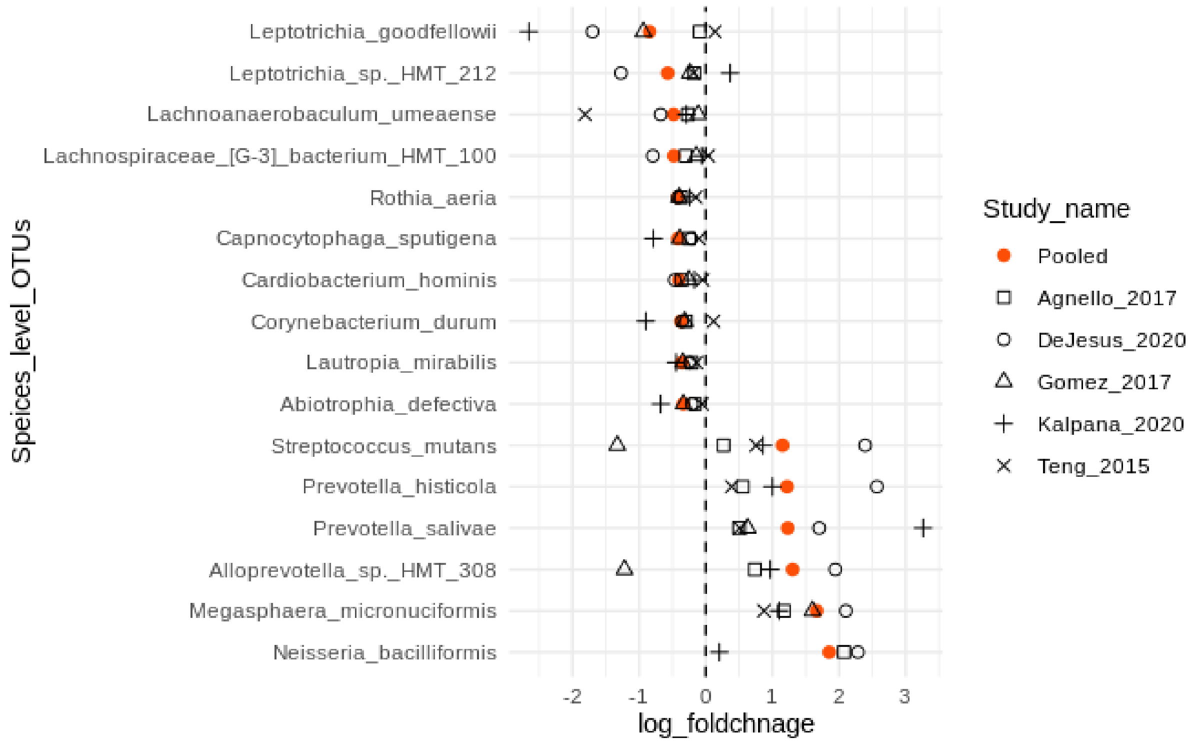

Figure S1: **Differentially abundance taxa on raw abundance without batch correction values at species-level using DESeq2, related to Figure 7.** Only the significant taxa in Pooled studies were selected for plotting.

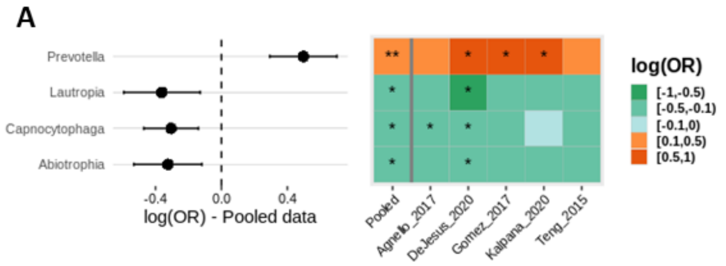

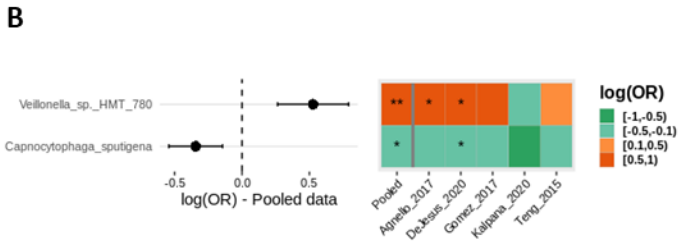

Figure S2: **Meta-analysis for differentially abundance taxa without batch correction values, related to Figure 7.** (A) Genus-level. (B) Species-level. The heatmap represents the log odd ratios of differentially abundant with significant p-adjusted value (p-adjusted<0.05) in pooled dataset along with the odd ratio estimates in each dataset. The forest plot signifies 95% confidence interval for the log odd ratio values for each taxon in pooled dataset.

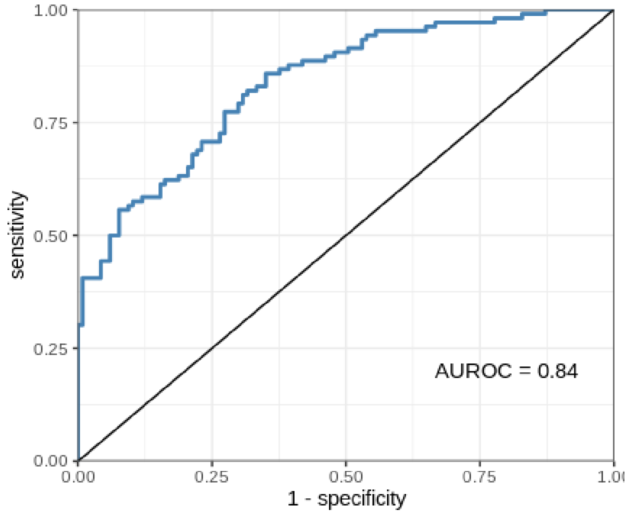

Figure S3: **Random Forest performance in terms of AUROC with combined genus and species-level OTUs for Pooled dataset with batch-corrected values, related to Figure 9.**

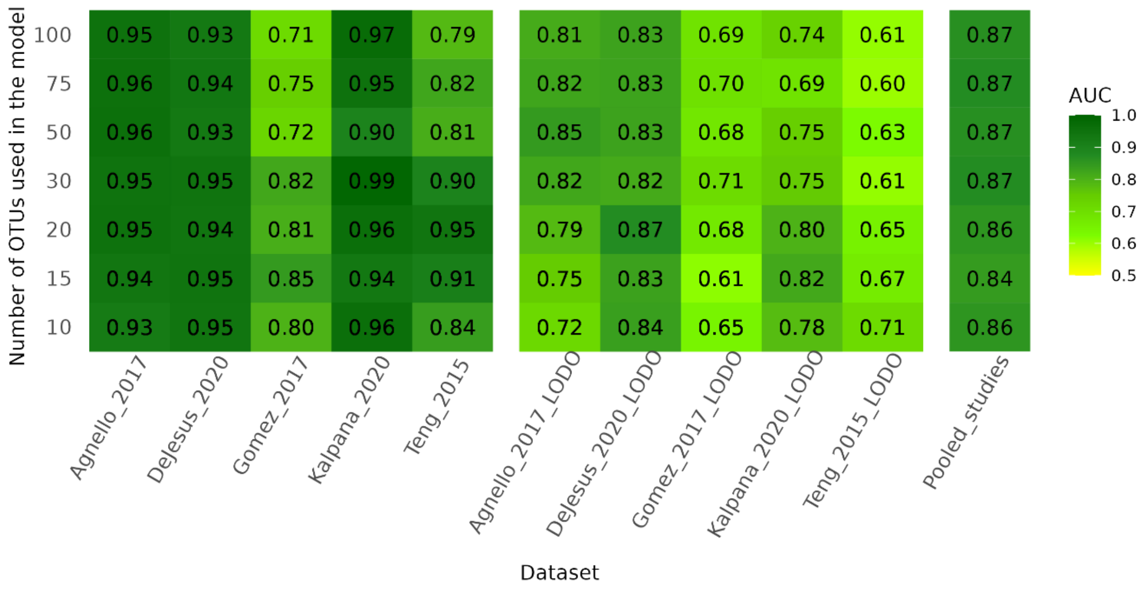

Figure S4: **AUROC values with species-level data with CLR normalization without batch correction, related to Figure 9**. The left panel represents the cross-validation results for each dataset. The middle panel is for model performance for LODO analysis. In LODO analysis, all datasets except one were used for the training and the left-out dataset was then used for the testing to assess the generalizability of the model. The rightmost column is for the cross-validation performance of the pooled dataset. The y-axis represents the number of top OTUs used for model assessment.

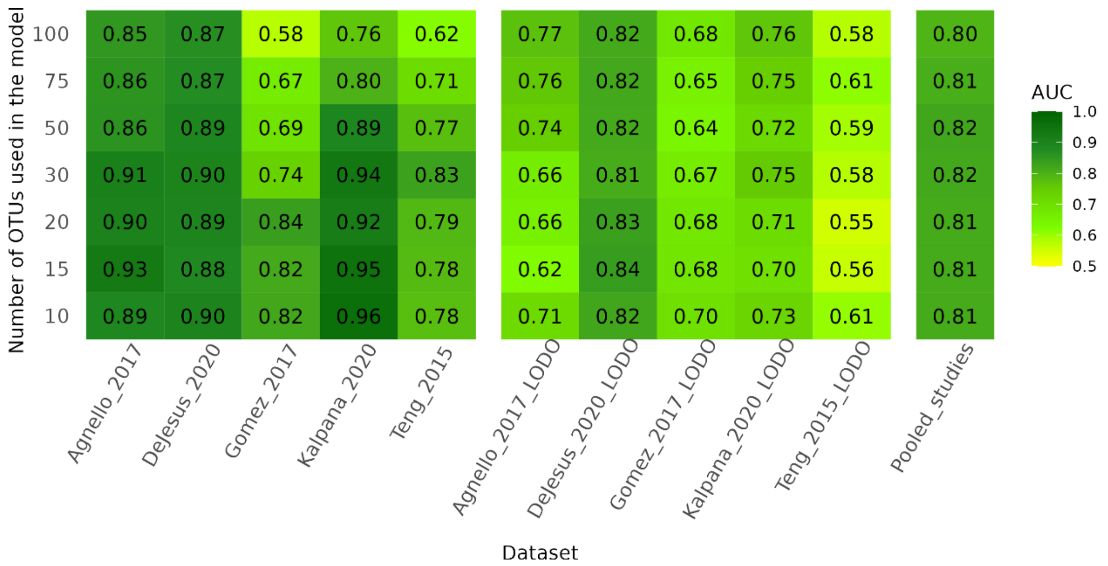

Figure S5: **AUROC values with batch corrected values using genus-level data, related to Figure 9.** The left panel represents the cross-validation results for each dataset. The middle panel is for model performance for LODO analysis. In LODO analysis, all datasets except one were used for the training and the left-out dataset was then used for the testing to assess the generalizability of the model. The rightmost column is for the cross-validation performance of the pooled dataset. The y-axis represents the number of top OTUs used for model assessment.

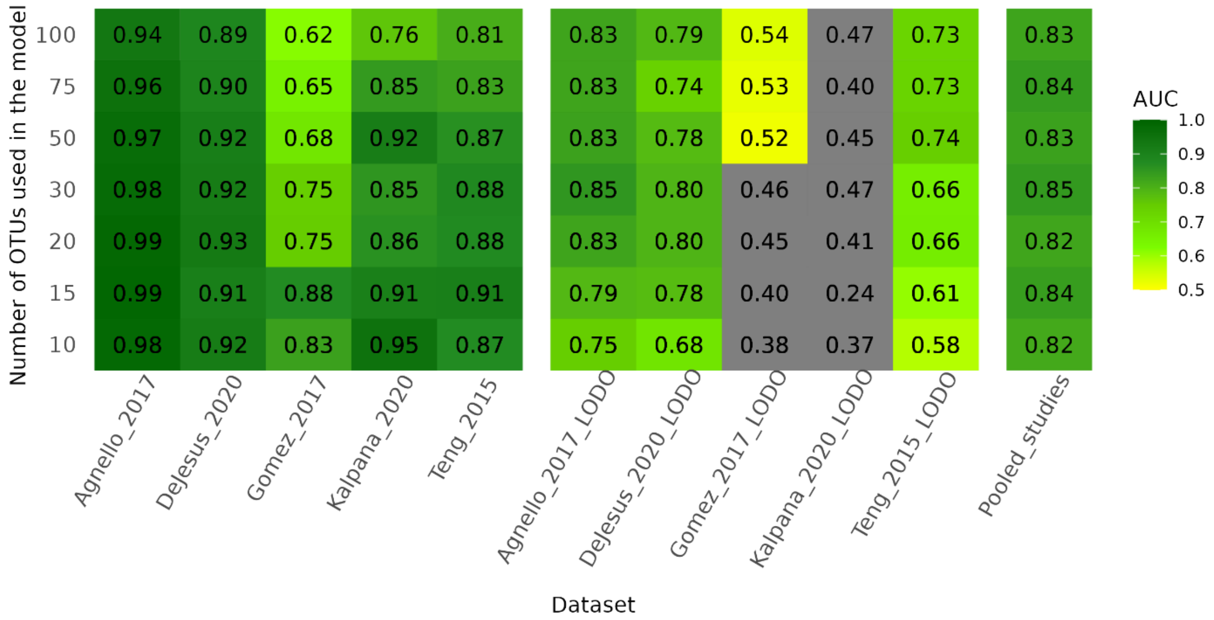

Figure S6: **AUROC values with imputed data with CLR normalization with species-level data, related to Figure 9.** The left panel represents the cross-validation results for each dataset. The middle panel is for model performance for LODO analysis. In LODO analysis, all datasets except one were used for the training and the left-out dataset was then used for the testing to assess the generalizability of the model. The rightmost column is for the cross-validation performance of the pooled dataset. The y-axis represents the number of top OTUs used for model assessment.
